# Identification of Proteins Deregulated by Platinum-Based Chemotherapy as Novel Biomarkers and Therapeutic Targets in Non-Small Cell Lung Cancer

**DOI:** 10.3389/fonc.2021.615967

**Published:** 2021-03-11

**Authors:** Sarah-Louise Ryan, Keyur A. Dave, Sam Beard, Martina Gyimesi, Matthew McTaggart, Katherine B. Sahin, Christopher Molloy, Neha S. Gandhi, Eric Boittier, Connor G. O’Leary, Esha T. Shah, Emma Bolderson, Anne-Marie Baird, Derek J. Richard, Kenneth J. O’Byrne, Mark N. Adams

**Affiliations:** ^1^ Faculty of Health, School of Biomedical Sciences, Institute of Health and Biomedical Innovation, Translational Research Institute, Queensland University of Technology, Woolloongabba, QLD, Australia; ^2^ Faculty of Science and Engineering, School of Chemistry and Physics, Institute of Health and Biomedical Innovation, Queensland University of Technology, Brisbane, QLD, Australia; ^3^ Cancer Services, Princess Alexandra Hospital, Woolloongabba, QLD, Australia; ^4^ Department of Clinical Medicine, Trinity Translational Medicine Institute, Trinity College Dublin, Dublin, Ireland; ^5^ Thoracic Oncology Research Group, Labmed Directorate, St. James’s Hospital, Dublin, Ireland

**Keywords:** non-small cell lung cancer, platinum-based chemotherapy, cisplatin, quantitative proteomics, biomarkers, therapeutic targets, machine learning

## Abstract

Platinum-based chemotherapy remains the cornerstone of treatment for most people with non-small cell lung cancer (NSCLC), either as adjuvant therapy in combination with a second cytotoxic agent or in combination with immunotherapy. Resistance to therapy, either in the form of primary refractory disease or evolutionary resistance, remains a significant issue in the treatment of NSCLC. Hence, predictive biomarkers and novel combinational strategies are required to improve the effectiveness and durability of treatment response 6for people with NSCLC. The aim of this study was to identify novel biomarkers and/or druggable proteins from deregulated protein networks within non-oncogene driven disease that are involved in the cellular response to cisplatin. Following exposure of NSCLC cells to cisplatin, *in vitro* quantitative mass spectrometry was applied to identify altered protein response networks. A total of 65 proteins were significantly deregulated following cisplatin exposure. These proteins were assessed to determine if they are druggable targets using novel machine learning approaches and to identify whether these proteins might serve as prognosticators of platinum therapy. Our data demonstrate novel candidates and drug-like molecules warranting further investigation to improve response to platinum agents in NSCLC.

## Introduction

Lung cancer is a significant health care burden, accounting for 18.4% of all cancer-related deaths (1). The most commonly diagnosed form of this disease is non-small cell lung cancer (NSCLC) constituting 85% of all cases. Treatment options for NSCLC include front-line immune checkpoint inhibitors as monotherapy, particularly in patients with programmed death receptor ligand (PD-L1) expression in >50% of tumor cells detected by immunohistochemistry (2), or in combination with platinum-based chemotherapy in the advanced setting (2–4), maintenance immunotherapy for unresectable stage III disease after radical chemoradiotherapy (5) or adjuvant platinum-based chemotherapy for those with high risk resected early stage NSCLC (6). However, while survival rates are improving with advances such as screening, improved imaging technology and surgical techniques along with the use of in targeted therapeutics and immunotherapy agents (7), 5-year survival rates remain poor at 20.5% (8).

Cisplatin, *cis*-Diammineplatinum (II) dichloride, is the most widely studied platinum agent (9–13), and is currently one of the most effective chemotherapeutic agents available for treating NSCLC. However, objective response rates for patients with advanced NSCLC remain poor at 40%–50% (14, 15). Even in combination with immunotherapy the objective response rates remain between 35% and 70% pending the level of PD-L1 expression (2, 5, 16). The development of refractory disease is primarily due to intrinsic or acquired drug resistance which reduces the effectiveness of platinum agents used alone or in combination during in the management of NSCLC (17, 18). Thus, clinical trials are ongoing to identify novel combination strategies for use with traditional chemotherapy (19). Likewise, patients with resected stage II or III disease receive adjuvant platinum-based chemotherapy with the aim of reducing risk of future recurrence. In this setting, meta-analysis data suggest a benefit in terms of survival of 5% at 5 year with the addition of chemotherapy after a successful complete surgical resection. Many patients will still experience disease recurrence after this therapy suggesting a disease biology that is refractory to chemotherapy (6). Further strategies are warranted to amplify/complement the effects of platinum-based chemotherapy.

The recognized mechanism of action for cisplatin is *via* the induction of DNA damage. Cisplatin induces anti-tumor effects by predominantly binding to DNA or RNA to form DNA-platinum adducts (Stordal & Davey, 2007). These intra- or inter-strand crosslinks (ICLs) induce DNA damage response pathways by blocking the unwinding of double stranded DNA which is necessary for the cellular processes of transcription and DNA replication. Pathways implicated in adduct removal include the base excision and nucleotide excision repair (BER and NER) pathways and Fanconi anemia (FA) pathway (20, 21). The extent of damage or failure to remove these blockages and repair damaged DNA account for the cytotoxic effects of cisplatin resulting in tumor cell death. The DNA damage response (DDR) is also associated with resistance to cisplatin, with upregulation of these pathways promoting repair of DNA adducts and enabling tumor cell survival (22–24).

Alongside the DDR, over 147 mechanisms of platinum resistance have been suggested (25). These varied and complex mechanisms also include reduced uptake or increased drug efflux *via* copper transporters to reduce intracellular drug concentrations yielding acquired resistance (26–32). Recent unbiased screening (33–36) has highlighted platinum resistance in lung cancer arises *via* predominant cellular programs of the aforementioned DNA repair as well as upregulating expression of cell cycle-associated genes, transforming growth factor (TGF)-β signaling and apoptosis avoidance (22). Hence, blocking or exploiting the function of proteins within these pathways and cellular programs might serve as a useful approach to improve the sensitivity of tumors to platinum agents.

Novel strategies to enhance platinum-based therapy response rates will rely upon identifying those people with NSCLC who will best respond to platinum-based therapy, as well as providing alternate treatment options for those less likely to benefit from platinum-based regimens. As such, employing predictive biomarkers and introducing synergistic therapies might improve the effectiveness of therapy by reducing unnecessary treatment or doses of therapy. The aim of this study was to identify protein networks involved in the cellular response to cisplatin by mining quantitative mass spectrometry data. Our study focused on cisplatin response in non-oncogene driven disease where platinum-based chemotherapy is employed in the absence of targeted therapies. We sought to examine whether these deregulated proteins identified in our study were druggable using novel machine learning approaches and to identify whether these proteins might serve as prognosticators of platinum therapy. Based upon our approach, these data demonstrate there are several novel protein candidates and drug-like molecules that warrant further investigation. With additional preclinical evaluation and future clinical investigations, our findings may lead to potentially improved responses to platinum agents.

## Materials and Methods

### Antibodies and Reagents

Antibodies against aldehyde dehydrogenase 3A1 (ALDH3A1); ab76976) and TP53I3 (ab64798) were purchased from Abcam. The α-tubulin antibody (T9026) was purchased from Sigma Aldrich. Donkey anti-rabbit and anti-mouse Alexa Fluor 680 and 800 antibodies were purchased from Life Technologies. Complete EDTA-free protease inhibitor mixture was from Roche Applied Sciences and phosphatase inhibitor cocktail Cell Signaling Technology. *cis*-Diammineplatinum (II) dichloride (cisplatin) was purchased from Merck.

### Cell Culture and Treatment

All cell lines were purchased from the American Type Culture Collection (ATCC) and maintained at 37°C in a humidified atmosphere containing 5% CO_2_ in RPMI 1640 medium supplemented with 1% L-Glutamine and 1% non-essential amino acids (NEAA). Cell culture media were supplemented with 10% (v/v) heat-inactivated fetal bovine serum (FBS).

For differential mass spectrometry-based quantitative proteomics, H460 cells were treated cisplatin (7.5 µM) for 24 h. For *in vitro* drug treatments prior to western blot analysis, cells were treated with cisplatin (5 µM) for 12 h.

### Nano Liquid Chromatography Mass Spectrometry Analysis

MS analysis was performed using an AB Sciex 5600+ TripleTOF mass spectrometer interfaced to an Ekspert™ NanoLC system. Sample preparation and library construction for differential quantitative liquid chromatography (LC)-MS/MS was performed and analyzed as previously described (37). Briefly, equal amounts of sample in triplicate were prepared by filter-aided sample preparation (FASP) (38) and tryptic digested peptides were analyzed by LC-MS/MS. For each biological replicate, a spectral library was generated with a traditional data- dependent acquisition (DDA) approach. The DDA data was searched against a human proteome library (UP000005640; UniProt.org) using ProteinPilot 5.0 (SCIEX). Following generation of the spectral library, the identification and quantification of cisplatin-treated and untreated H460 triplicate cell lysate derived peptides were quantified using an LC-MS/MS approach known as variable sequential window acquisition of all theoretical mass spectra (SWATH-MS) with a targeted data extraction strategy to mine the resulting fragment ion data set. SWATH-MS transition, peptide, and protein level summarization was performed using Skyline (39) by normalization at the peptide level using median normalization and Tukey median polish for summarization over all features in a run.

Log2 transformation was performed prior to further statistical analysis. Differentially regulated proteins were identified by applying empirical Bayes moderated t-statistics tests (40, 41) implemented in the limma package using R statistical environment (version 3.5.2) and the Benjamini-Hochberg correction (42) was applied to control the false discovery rate [FDR (or q-value)]. List of all quantified proteins (including differentially regulated proteins) by LC-MS/MS are listed in **Supplemental Table 1**.

### Functional Analysis

Statistically significant proteins from the comparison of cisplatin treated cells versus untreated cells at a q-value threshold of ≤ 0.1 (65 proteins) were used for gene ontology (GO), Kyoto encyclopedia of genes and genomes (KEGG) and Pathway over representation analysis. For protein network analysis, all proteins at a q-value threshold ≤0.1 were selected for StringDB analysis. Network was generated with default confidence (medium confidence, 0.4) and evidence from active interaction sources included, text mining, experiments, databases, co-expression, neighborhood, gene fusion, and co-occurrence.

Signaling network analysis was conducted using Ingenuity^®^ Pathway Analysis (IPA) (QIAGEN). Right-tailed Fisher’s exact test was used to determine over representation probability of canonical pathways in the protein dataset. Z-score analysis was also performed in IPA to predict activation or inhibition of significant canonical pathways based on Log fold-change values of the input molecules from the quantitative proteomics data set. All IPA canonical pathways are listed in **Supplemental Table 2**.

GO biological processes and KEGG enrichment analyses were performed using the ClueGo (ver. 2.5.6) (43) plugin in Cytoscape (ver. 3.7.2). Functionally grouped and annotated networks were assessed by hypergeometric testing (enrichment/depletion, two-sided hypergeometric test and Bonferroni step down correction). Networks were visualized using the default ClueGo layout with functionally grouped network, GO/KEGG terms as nodes and linked based on their kappa score level. Percentage of mapped genes belonging to each term were also represented. Node size is representative of the term enrichment significance. A kappa coefficient of 0.4 was used as a threshold values while redundant groups with >50% overlap were merged.

### Collection of Lysates and Western Blot Analysis

Prior to western blot analysis, cells were washed once with ice-cold phosphate buffered saline and lysed in lysis buffer (50 mM HEPES (pH 7.5), 150 mM KCl, 5 mM EDTA, 0.05% IGEPAL CA-630 (v/v), 1x protease inhibitor cocktail and 1x phosphatase inhibitor cocktail). Lysates were briefly sonicated before low speed centrifugation (900 g, 10 min at 4°C) with total protein concentration determined by Bicinchoninic Acid (BCA) protein assay. Equal amounts of lysate were denatured in 1x Laemmli buffer containing 8% β-mercaptoethanol by heating for 5 min at 80°C. Samples were subjected to SDS-PAGE analysis using Bolt 4%–12% Bis-Tris Plus pre-cast gels (Life Technologies) before protein transfer to nitrocellulose membrane (GE Healthcare Life Sciences) using the Novex system (Life Technologies). Membranes were blocked in Odyssey blocking buffer (Li-Cor Biosciences) and incubated with primary antibodies overnight at 4°C with gentle agitation. All antibodies were used at a 1:1,000 dilution with the exception of the anti-α-tubulin antibody (1:5,000). Membranes were washed with PBS containing 0.1% Tween-20, incubated with appropriate secondary antibodies and scanned using an Odyssey CLx imaging system (Li-Cor Biosciences). Images were subjected to densitometric analysis using ImageJ software.

### Prediction of Druggable Proteins

The druggability and developability space is rapidly evolving in the post-structural genomics era. The development of computational methods using sequence, protein structure and ligand (including Lipinski's rule of five) has helped characterize protein targets and describe strategies for the optimal integration of protein druggability data (44). As an initial step, a recently described machine learning prediction model was utilized for the extrapolation of potentially druggable proteins from the upregulated protein list (45). This machine learning approach was derived from a set of 666 known druggable as well as 219 non-druggable protein sequences. In our study, all sequences for up and downregulated proteins identified by quantitative mass spectrometry were collected in FASTA format. Druggable features of these proteins were evaluated using available python scripts (https://github.com/muntisa/machine-learning-for-druggable-proteins). Briefly, these scripts applied thirteen machine learning classification types which were used to learn and recognize characteristic traits for the prediction and classification of proteins into druggable categories. Prediction performance was evaluated against several breast cancer proteins, cancer-driving proteins and RNA-binding proteins. Druggable and non-druggable sequences were given the labels 1 and 0 respectively; therefore, scores closest to 1 are proteins with greatest druggable potential and are more likely to be a promising drug target candidate. This approach allows unbiased prediction of proteins that are less studied with limited information about their function. Moreover, this approach can be applied to predict druggability of proteins with unresolved or incomplete structure which would otherwise affect other approaches such as the structure-based druggability prediction method.

To complement the machine learning modelling, two additional approaches were used to assess protein structural druggability potential and identify compounds known to target the deregulated proteins. First, proteins with three-dimensional models and ligandable cavities were evaluated using the CanSAR knowledgebase (46) to confirm druggability potential. The previous studies revealed that cancer proteins tend to interact more strongly than other categories of proteins including essential and control proteins in the human interactome (47), therefore CanSAR network-based protein-protein interactions data can serve as potential therapeutic targets in cancer among certain important pro-carcinogenic signaling pathways. The Binding Database was also examined to identify investigational and/or approved chemical entities from the literature for a druggable target (48). All identified compounds are listed in **Tables 1** and **2** with additional compounds and structures listed in **Supplemental Table 3**.

**Table 1:** Druggability assessment of upregulated proteins.

Gene name	Uniprot	PDB codes*	Druggability score**	Ligands***	Patent	CanSAR protein interactome (Number of proteins in the signaling network)
DPYSL2	Q16555	5YZ5	0.999999953	Erlosamide	563KS2PQY5	146 interactions with 104 interactors
ALDH3A1	P30838	3SZA 4H80 4L1O 4L2O	0.99999987	Benzimidazole Analogues and diarylamine derivatives such as CB7, CB29 (see **Supplemental Table 3**)	US9320722 (2016)	38 interactions with 26 interactors
PAPSS2	O95340	24X4	0.999999683	Indazole Analogues	N/A	40 interactions with 35 interactors
FDXR	P22570	N/A	0.999999672	N/A	N/A	61 interactions with 39 interactors
CTSL	P07711	1ICF 1MHW 2YJB 3H8B	0.999999086	Acyclic cyanamide-based inhibitors, Azadipeptide Nitriles, etc. (see **Supplemental Table 3**)	N/A	128 interactions with 87 interactors
ITGB1	P05556	3VI4 4WK2 3T9K	0.999999059	N/A	N/A	468 interactions with 307 interactors
KRT8	P05787	N/A	0.999986795	N/A	N/A	207 interactions with 146 interactors
TFRC	P02786	3S9L 3S9M 3S9N 1DE4 3KAS 6D03 6D04 6D05	0.997152835	N/A	N/A	244 interactions with 187 interactors
TP53I3	Q53FA7	2J8Z 2OBY	0.967769972	N/A	N/A	23 interactions with 13 interactors
NME1	P15531	1JXV 1UCN 2HVD 2HVE	0.945227623	N/A	N/A	192 interactions with 148 interactors

*PDB codes obtained from CanSAR database for a given sequence with maximum coverage and ligand pockets.

**Obtained from machine learning approach of Lopez-Cortes et al.

***Source, BindingDB and/or CanSAR. Approved and investigational drugs details were extracted from CanSAR whereas chemical entities reported in the literature were compiled from BindingDB.

**Table 2:** Druggability assessment of downregulated proteins.

Gene name	Uniprot	PDB codes*	Druggability score**	Ligands***	Patent	CanSAR protein interactome (Number of proteins in the signaling network)
NUDC	Q9Y266	N/A	0.999999993	N/A	N/A	350 interactions with 282 interactors
RACK1	P63244	4AOW 4D5L 4D61 4KZX 4KZY 4KZZ	0.99999981	N/A	N/A	424 interactions with 291interactors
CLUH	O75153	N/A	0.999999771	N/A	N/A	93 interactions with 74 interactors
HNRNPAB	Q99729	N/A	0.999999747	N/A	N/A	191 interactions with 144 interactors
AARS	P49588	4XEM 4XEO 5V59 5KNN	0.999999059	Thiazole derivatives (see **Supplemental Table 3**)	N/A	332 interactions with 205 interactors
UCHL5	Q9Y5K5	3RIS 4UEL 4UF5 4WLP	0.999999059	4-hydroxycyclohexanone scaffolds (see **Supplemental Table 3**)	N/A	374 interactions with 220 interactors
RPL7	P18124	N/A	0.999990505	N/A	N/A	630 interactions with 335 interactors
RPL29	P47914	N/A	0.999988489	N/A	N/A	442 interactions with 230 interactors
RPL27A	P46776	N/A	0.999986776	N/A	N/A	517 interactions with 259 interactors
YARS	P54577	1N3L 4Q93 4QBT 5THL 5THH 1NTG	0.99745549	Glutamic acid esters wherein the alcohol moiety is ribose, prolinol or substituted piperidines (see **Supplemental Table 3**)	N/A	90 interactions with 53 interactors

*PDB codes obtained from CanSAR database for a given sequence with maximum coverage and ligand pockets.

**Obtained from machine learning approach of Lopez-Cortes et al.

***Source, BindingDB and/or CanSAR. Approved and investigational drugs details were extracted from CanSAR whereas chemical entities reported in the literature were compiled from BindingDB.

### Bioinformatics and Survival Analysis

Survival analysis was performed on Illumina gene expression data and clinical information with the University of Texas Lung Specialized Program of Research Excellence (UT Lung SPORE) dataset and obtained from the GEO database (GSE42127) (49). Within this cohort, 127 patients were observed following curative resection (OBS) whereas 49 patients received adjuvant platinum-based chemotherapy (ACT). Illumina probes corresponding to the identified differentially regulated proteins are listed in **Supplemental Table 4**. High versus low transcript expression was stratified according to median expression with OBS and ACT overall survival curve comparison performed by the Kaplan-Meier method and log-rank test where *P*-value > 0.05 was considered significant.

## Results

### Cisplatin-Induced Regulation of NSCLC Proteins

**Figure 1:**
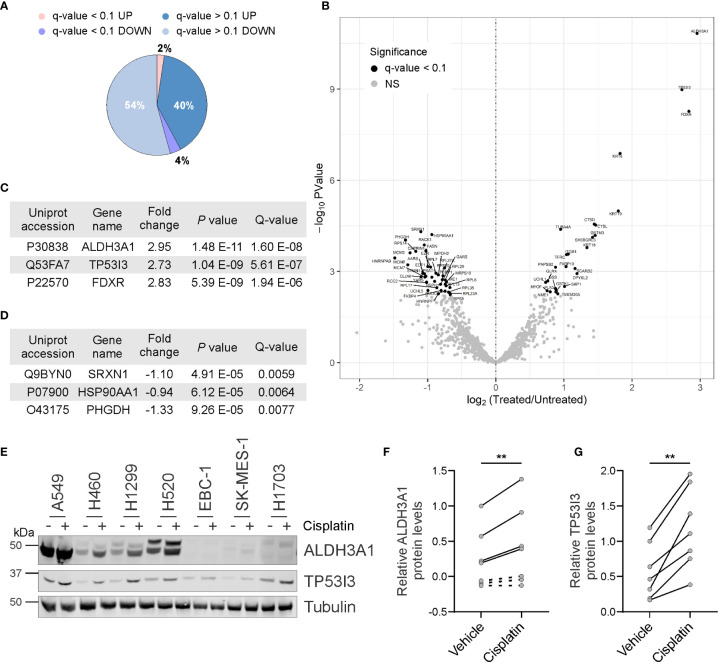
Identification of proteins deregulated by cisplatin exposure. **(A)** Pie chart showing proportion of identified proteins up or downregulated following cisplatin exposure and those deregulated proteins considered significant (q-value < 0.1). **(B)** Volcano scatter plot of log2 fold protein changes (cisplatin treatment versus untreated) ranked by significance (-log_10_ *P* value) with proteins of q-value < 0.1 highlighted in black. **(C)** List of top three cisplatin-induced upregulated proteins ranked by Q-value. **(D)** List of top three cisplatin-induced downregulated proteins ranked by Q-value. **(E)** Representative endogenous ALDH3A1 and TP53I3 western blot analysis from lysates of NSCLC cell lines treated in the absence or presence of cisplatin. α-Tubulin used as loading control. **(F)** Densitometry quantification of **(E)**, with dot points representing average log2 of relative ALDH3A1 levels from three independent experiments with lines connecting respective untreated and cisplatin treated cell lines (paired Student’s *t* Test, two tailed **P = 0.0081). **(G)** Densitometry quantification of **(E)**, with dot points representing average log2 of relative TP53I3 levels from three independent experiments with lines connecting respective untreated and cisplatin treated cell lines (paired Student’s *t* Test, two tailed **P = 0.0014).

To identify proteins that might be exploited to improve platinum response, we sought to identify proteins from NSCLC cell lines that were deregulated upon acute cisplatin exposure. We reasoned that inhibiting the function of these identified deregulated proteins which, while not considered classical targets, but may serve to promote cancer cell survival following cisplatin exposure, could improve therapy response. By following our earlier approach, the H460 NSCLC cell line was treated with the appropriate IC_50_ concentration of cisplatin. This cell line was selected as a commonly utilized non-squamous cell line for *in vitro* assays. Whole cell lysates were collected from untreated and cisplatin treated cells and subjected to quantitative SWATH-MS to identify differentially expressed proteins. Of the 1081 proteins robustly identified, 430 proteins were upregulated following cisplatin exposure, while 586 proteins were downregulated (**Supplemental Table 1**). A total of 65 differentially regulated proteins were statistically significant with a q-value of less than 0.1 (**Figure 1A**). As shown in **Figure 1B**, cisplatin exposure induced the upregulation of 26 of these proteins and the downregulation of 39 proteins. The top three proteins based on log2 fold change and FDR significance were the ALDH3A1, TP53I3 and ferredoxin reductase (FDXR) upregulated proteins (**Figure 1C**), whereas the top three downregulated proteins were sulfiredoxin-1 (SRXN1), heat shock protein 90α (HSP90AA1) and phosphoglycerate dehydrogenase (PHGDH) (**Figure 1D**).

Based on the SWATH-MS differentially regulated protein candidates, we sought to orthogonally validate our findings from the single H460 cell line by using western blot analysis and a wider panel of NSCLC cell lines that were treated with or without cisplatin. The protein levels of ALDH3A1 and TP53I3 were selected for examination by western blot analysis given our access to commercially available antibodies and that these proteins were identified as significantly upregulated by cisplatin. Consistent with the SWATH-MS, cisplatin significantly induced the upregulation of ALDH3A1 in all cell lines where the protein was detectable (*p* = 0.0081; **Figures 1E, F**). Notably, ALDH3A1 expression was not detected in the squamous cell carcinoma cell lines EBC-1, SK-MES-1, and H1703. Consistently, protein levels for TP53I3 were also significantly upregulated following cisplatin exposure in all cell lines evaluated (*p* = 0.0014; **Figures 1E, G**). Taken together, these data corroborate with the differential global quantitative MS profile obtained using H460 treated cells and suggest, that at least for ALDH3A1 and TP53I3, modulation of these protein levels are a common *in vitro* response to cisplatin exposure.

### Comparative Pathway Analysis

**Figure 2:**
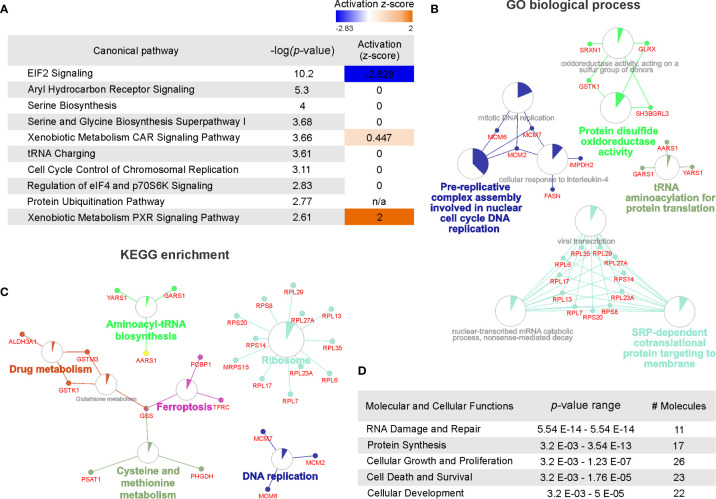
Pathway overrepresentation and gene ontology analysis of cisplatin-induced significantly deregulated proteins. **(A)** Top 10 canonical pathways identified by Ingenuity pathway analysis associated with cisplatin response ranked by significance (-log_10_ *P* value) and predicted pathway activation or inhibition indicated by Z-score. **(B)** Gene ontology enrichment analysis assessed by Gene ontology (GO) biological processes with functional networks visualized using ClueGo. GO biological process terms listed as nodes with genes belonging to each term represented. Node size represents the term enrichment significance. **(C)** Kyoto encyclopedia of genes and genomes (KEGG) enrichment analyses assessing gene ontology with functional networks visualized using ClueGo. KEGG terms listed as nodes with genes belonging to each term represented. Node size represents the term enrichment significance. **(D)** Top five molecular and cellular functions identified by Ingenuity pathway analysis (IPA) ranked by *P* value range and listing the number of molecules identified.

To examine the functional importance of deregulated proteins following cisplatin exposure, all *q*-value ≤ 0.1 proteins were subjected to STRINGdb analysis. This database was examined to evaluate potential protein-protein interactions between identified proteins (50). These data indicated there to be clusters of networks existing between 77% of the significantly deregulated proteins following cisplatin treatment (**Supplemental Figure 1**). Next, we sought to identify the function for these proteins by performing pathway analysis using IPA. A total of 141 canonical pathways were identified (**Supplemental Table 2**) with 39 of these pathways reaching the significance threshold (*p* < 0.05; **Supplemental Table 2**). Of the top 10 canonical pathways ranked by significance, an activation z-score was calculated for three pathways (**Figure 2A**). EIF2 signaling was predicted to be inhibited following cisplatin exposure (z-score = -2.83) whereas xenobiotic metabolism was predicted to be activated in response to cisplatin (constitutive androstane receptor (CAR) signaling and pregnane X receptor (PXR) signaling pathways with respective z-scores of 0.45 and 2.0). These findings are consistent with the observations that cisplatin inhibits eukaryotic translation (51) and, like other chemotherapeutic agents, induces the upregulation of drug metabolism pathways (52, 53).

The cisplatin-induced differentially regulated proteins were also classified using over representation analysis to enrich for clusters based on protein ontology. As shown in **Figure 2B**, protein function identified by GO biological process indicated predominant clusters involved in DNA replication, disulphide oxidoreductase activity, tRNA biosynthesis and signal-recognition particle (SRP)-dependent regulation of translation. To further evaluate biological function of the differentially regulated proteins, we also applied KEGG enrichment analysis. As shown in **Figure 2C**, several pathways were identified consistent with the GO analysis including ribosome regulation, DNA replication and tRNA biosynthesis. In addition, protein clusters involved in ferroptosis, drug metabolism and cysteine and methionine metabolism pathways were identified. Supporting the GO and KEGG enrichment analysis, IPA analysis of cellular and molecular function identified the top pathway functions as RNA damage and repair, protein synthesis and pathways involved in regulating cellular proliferation, survival and development (**Figure 2D**). Notably, IPA analysis also identified p53 as the top upstream regulator linking this protein with modulation of tumor cell proliferation (**Supplemental Figure 2**). Taken together, the top cisplatin-induced differentially regulated proteins modulate translation, replication, pathways exposure induced pathways regulating DNA damage, translation and metabolic control.

### Identification of Druggable Proteins

Having identified biological pathways that are impacted in response to cisplatin exposure, we postulated that the significantly differentially regulated proteins might prove exploitable as potential therapeutic targets to enhance chemotherapy sensitivity. The differentially regulated proteins were separated into those proteins upregulated (**Table 1**) or downregulated (**Table 2**) in response to cisplatin exposure. We applied a recently reported machine learning approach (45) to identify which proteins could be inhibited with drugs/ligands by having possible targetable regions within the protein based upon their protein sequence. This approach predicts druggable proteins by analyzing the primary protein sequence with confidence scores close to 1.0 considered druggable. The top 10 predicted druggable targets identified as significantly upregulated by our proteomics are listed in **Table 1**. Validating the predictive machine learning approach, structural ligandability was also explored based on CanSAR database. Structural ligandability refers to identification of protein pockets with the 3D structures of target proteins or domains and are summarized in **Supplemental Table 3**. Of these proteins, structures of full-length or certain domains have been solved, for eight proteins. Besides machine learning and structural ligandability, we also compiled ligand information from CanSAR and BindingDB for these targets wherein four of the top 5 proteins have identified compounds which target these proteins. For example, the anti-seizure drug lacosamide is indicated to specifically bind dihydropyrimidinase-related protein 2 (DPYSL2) (54). Patent literature also exists for the drugs identified that target the proteins DPYSL2 and ALDH3A1. We have provided datasets which includes structure, literature reference and activity data compiled from BindingDB as supporting information for the top ranked up- and downregulated druggable proteins.

As shown in **Table 2**, the top 10 predicted druggable targets were also identified for the proteins downregulated in response to cisplatin exposure. Like the upregulated proteins, all but four of the proteins predicted to be druggable had a solved protein structure. Moreover, compounds have been identified that target three of the top 10 proteins, alanyl-tRNA synthetase (AARS), ubiquitin carboxy-terminal hydrolase L5 (UCHL5) and tyrosyl-tRNA synthetase (YARS). Unlike the upregulated proteins, these compounds do not have patent coverage. Besides the protein structure and ligand based approaches, protein-protein interactions form signaling nodes and hubs to transmit pathophysiological cues along molecular networks, thereby promoting cancer progression, invasion, and/or metastasis (55). We report the number of signaling proteins in the interactome curated from CanSAR (**Tables 1** and **2**) for the up and downregulated proteins. Disruption of protein-protein interactions is critical for cancer and offers a novel and effective strategy to curtail the transmission of oncogenic signals. Taken together, by employing these machine learning, structure and ligand-based approaches, we have identified novel therapeutic targets that are regulated by cisplatin. Further investigation is required to identify specific and potent drug-like molecules with therapeutic potential and determine the combinational potential of these compounds with chemotherapy.

### Prognostic Benefit of Cisplatin-Induced Deregulated Factors

**Figure 3:**
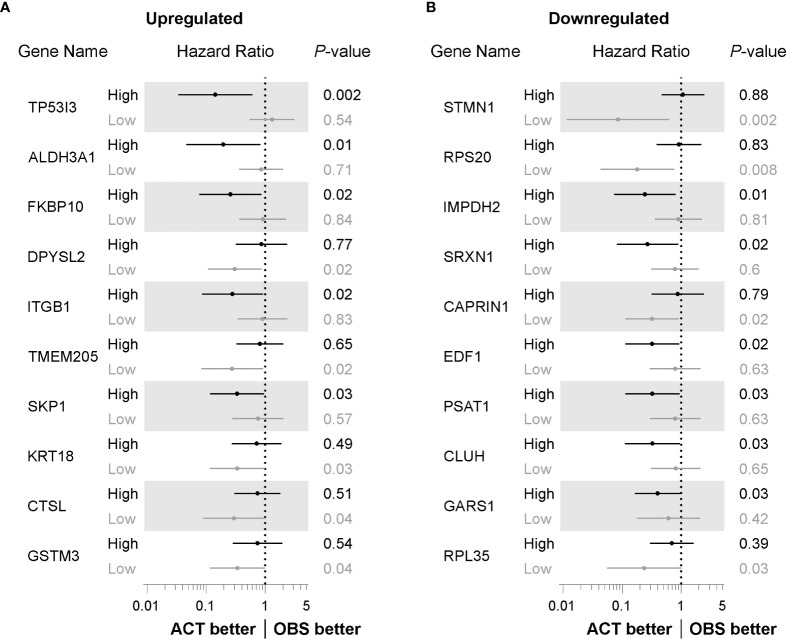
Identification of factors with prognostic benefit for treatment of NSCLC patients with adjuvant chemotherapy. **(A)** Forest plot showing hazard ratio (HR) and 95% confidence interval (CI) determined from overall survival Kaplan-Meier analysis of transcripts for proteins identified as upregulated following cisplatin exposure. Cases stratified by median transcript expression and compared according to those receiving adjuvant chemotherapy (ACT) or observation alone (OBS) and ranked *P* value determined by log-rank method. **(B)** Forest plot determined from Kaplan-Meier analysis as in **(A)**, comparing ACT versus OBS patients stratified by median expression of transcript for proteins identified as significantly downregulated following cisplatin exposure.

We next sought to determine whether the proteins identified as differentially regulated had prognostic benefit to identify people with NSCLC who would benefit from platinum-based chemotherapy. To investigate prognostic potential, we examined transcript expression for each significantly differentially regulated protein across microarray profiling of 176 evaluable NSCLC cases from the UT Lung SPORE cohort (GEO database GSE42127). In this cohort, NSCLC cases underwent curative resection and either received adjuvant platinum-based chemotherapy (ACT) or observation (OBS) alone. Cases were stratified based on median expression for each gene and survival analysis performed using the Kaplan-Meier method. The top 10 transcripts of upregulated proteins ranked by prognostic significance are listed in **Figure 3A**. Elevated levels of four of the top five prognostic transcripts had a hazard ratio (HR) of less than one suggesting that in these cases, patients benefited from adjuvant chemotherapy (TP53I3, ALDH3A1, FK506 binding protein 10 (FKBP10) and integrin beta-1 (ITGB1); **Figure 3A**). Interestingly, lower levels of five of the top 10 transcripts, including DPYSL2, transmembrane protein 205 (TMEM205), cytokeratin-18 (KRT18), cathepsin L (CTSL) and glutathione S-transferase M3 (GSTM3), were prognostic for patients treated with adjuvant chemotherapy Of the upregulated proteins, elevated TP53I3 levels were the most significant prognostic transcript (HR = 0.41, 95% CI: 0.03–0.59, *P* = 0.002) with adjuvant chemotherapy having little benefit in those cases with lower levels (HR = 1.31, 95% CI: 0.55–3.11, *P* = 0.54).

The prognostic benefit for transcripts of those proteins downregulated by cisplatin exposure was also investigated. As shown in **Figure 3B**, lower levels of four of the top 10 prognostic transcripts, including stathmin (STMN1), 40S ribosomal protein S20 (RPS20), caprin-1 (CAPRIN1) and 60S ribosomal protein L35 (RPL35), were associated with patients that benefited from adjuvant chemotherapy with a HR value of less than one. Elevated transcripts of inosine-5′-monophosphate dehydrogenase 2 (IMPDH2), SRXN1, endothelial differentiation-related factor 1 (EDF1), phosphoserine aminotransferase (PSAT1), Clu1/CluA homologue (CLUH) and glycyl-tRNA synthetase 1 (GARS1) were significantly associated with patients that benefited with adjuvant chemotherapy. Of the downregulated proteins, lower transcript levels of STMN1 had the most significant prognostic benefit for patients undergoing adjuvant platinum-based chemotherapy (HR = 0.08, 95% CI: 0.01–0.62, *P* = 0.002). Taken together, by assessing the prognostic value for those differentially regulated proteins, we have identified transcripts for these proteins that, at least in this patient cohort, have biomarker potential for improving platinum-based chemotherapy response.

## Discussion

Novel therapeutic strategies combined with predictive biomarkers of platinum-based chemotherapy response are needed to transform the clinical management of NSCLC. Such strategies are key to selecting the right patients for platinum-based chemotherapy and for overcoming therapy resistance which continues to affect health outcomes for people with this disease. We sought to identify potentially exploitable proteins that have a biological function in response to cisplatin exposure. Although not mutated and considered classically targetable such mutant epidermal growth factor receptor (EGFR), inhibiting the function of deregulated proteins could sensitize to platinum-based therapy. For this reason, we applied a SWATH-MS proteomic approach to identify exploitable proteins and biological pathways that are differentially regulated following cisplatin exposure. Previous screening using genetic-based approaches have confirmed mechanisms of action for cisplatin and its platinum analogues. For example, the anti-tumor effects of cisplatin and carboplatin result from the DNA damage response whereas oxaliplatin kills cells *via* ribosome biogenesis stress (56). Our quantitative analysis at the protein level in lung cancer revealed that alongside significant downregulation of ribosomal and translation control proteins, cisplatin negatively impacts DNA replication and amino acid synthesis while inducing upregulation of metabolic pathways. By further evaluating available clinical data and applying machine learning approaches, we identified potentially prognostic markers (**Figure 3**) and novel druggable targets (**Tables 1** and **2**) from the differentially regulated pathways which might prove actionable to enhance the effectiveness of platinum-based chemotherapy. While at an early exploratory stage, our study provides a platform for future preclinical evaluation. Our work will also complement ongoing and future drug discovery and biomarker development programs to ultimately improve future health outcomes for people with NSCLC.

Our proteomics analysis identified that cisplatin treatment yielded a marked downregulation of translation and protein synthesis. This is consistent with prior work indicating cisplatin, but not carboplatin or oxaliplatin negatively affect translation in *in vitro* systems (51). Of the proteins associated with translational control, five were found to be potentially druggable, potentially prognostic for chemotherapeutic response, or both: AARS, YARS, 60S ribosomal protein L7 (RPL6), 60S ribosomal protein L7 (RPL7), and 60S ribosomal protein L27a (RPL27a). AARS and YARS are involved in tRNA charging whilst RPL6, RPL7, and RPL27a are involved in EIF2 signaling for regulating both global and specific mRNA translation. AARS and YARS function to catalyze the alanine or tyrosine to tRNA attachment, respectively, in a two-step reaction: first by activating alanine or tyrosine *via* ATP to form Ala-AMP or Tyr-AMP, then transferring the activated alanine or tyrosine to tRNA. Not much is known about the role of AARS in lung cancer or any other solid malignancies. However, YARS is elevated in malignant gastric tissue compared to adjacent non-malignant tissue. The knockdown of YARS results in repressed proliferation and invasion in gastric cancer whilst enhancing apoptosis. The upregulation of YARS yields the reverse effects (57). RPL6, RPL7, and RPL27a are all components comprising the large ribosomal subunit. Increased expression of RPL6 promotes G1 to S phase transition of gastric cancer cells and leads to accelerated growth (58). RPL27a suppression leads to delayed tumorigenesis *in vivo (* 59). Whilst little is known about the involvement of RPL7 in lung cancer, it is involved in microsatellite instability in colorectal cancer (60). Taken together, although translation control is predicted to be inhibited following cisplatin, the malignant function for these proteins alongside our analysis suggest the identified proteins might serve as therapeutic options.

Further pathways identified by our analysis were proteins associated with cell death and survival. Of these proteins, HSP90AA1, ITGB1, cytokeratin-8 (KRT8) and receptor for activated C-kinase (RACK1) are identified prognostic markers for overall survival in solid malignancies including lung cancer. HSP90AA1 and KRT8 associate with a poorer rate of survival for people with NSCLC whereby higher protein expression is predictive of poorer prognosis and decreased survival (61, 62). Higher levels of KRT8 also significantly associate with tumor progression in NSCLC (62). ITGB1 and RACK1 are significant prognostic markers for survival in NSCLC and early-stage NSCLC, respectively (63, 64). These proteins are also prognostic markers in other solid malignancies. In gastric cancer, higher KRT8 expression also associates with poorer prognosis (65). Similarly, high ITGB1 expression associates with poor patient prognosis and independently correlates with shortened overall survival and shortened disease-free survival in people with colorectal cancer (66). In people with breast cancer, RACK1 is also a significant prognostic marker for survival (67). Indeed, our study identified elevated levels of ITGB1 as a significant prognosticator for overall survival in people treated with platinum-based therapy. Moreover, our findings indicate that ITGB1, alongside KRT8 and RACK1, have druggable potential. As such, further investigation is required to determine the diagnostic or therapeutic potential to improve platinum response.

Another key mechanism identified by our study is the predicted activation of cisplatin-induced drug metabolism. Our findings reveal the drug metabolism enzymes ALDH3A1, GSTM3 and glutathione S-transferase K1 (GSTK1) as significantly upregulated by cisplatin. Expression of drug detoxification and xenobiotic metabolizing enzymes are linked with patient response to chemotherapy and the onset of drug resistance by clearing reactive and cytotoxic compounds (52, 53). For example, ALDH3A1 activity is reported to increase upon cisplatin treatment, suggesting that this enzyme can metabolize cytotoxic aldehydes and confer resistance to cisplatin (68–70). Activity of glutathione transferases (GST), particularly GSTP1, is also associated with cisplatin sensitivity by detoxifying cisplatin to form an inactive cisplatin-glutathione conjugate (71, 72). Of the enzymes identified as deregulated, ALDH3A1 has druggable potential in NSCLC. While this might prove useful to improve platinum-based chemotherapy, targeting isoforms of the ALDH superfamily has been proposed for therapy resistant cancers (73). In addition, our findings also suggest stratifying patients according to ALDH3A1 or GSTM3 levels might prove beneficial. Indeed, in the case of GSTM3, a null genotype yielding low expression, is reportedly associated with reduced lung cancer risk (74). The enzymes identified in our study warrant further investigation in NSCLC as potential biomarkers and as druggable targets to improve patient chemotherapy response.

Our study has also identified the deregulated proteins CLUH and 3′-phosphoadenosine 5′-phosphosulfate (PAPS) synthetase 2 (PAPSS2) with other functional roles in metabolic regulation. CLUH is involved in mitochondrial biogenesis and distribution with genetic depletion of the protein resulting in loss of mitochondrial enzymes yielding oxidative phosphorylation defects and a dysfunctional Krebs cycle (75–77). Radio and chemotherapy resistant cancers, at least in triple negative breast cancer (78) and acute myeloid leukemia (79), are suggested to rely upon oxidative phosphorylation for survival and progression. As such, targeting CLUH or other proteins regulating oxidative phosphorylation might enhance sensitivity to therapy in lung cancer (80). The other protein we identified was PAPSS2. PAPSS2 is an isoform of PAPSS1, which synthesizes PAPS from ATP and inorganic sulphate (81, 82). While further investigation for PAPSS2 is required, depletion of the PAPSS1 isoform sensitized NSCLC to cisplatin suggesting that targeting sulfation reactions might improve activity of cisplatin or other DNA damaging agents (83). In line with these findings, our study indicated that both CLUH and PAPSS2 exhibited high confidence as potential drug targets.

While we have identified deregulated proteins as potential biomarkers or therapeutic targets with roles in drug metabolism, translational control, cell death, and survival, and DNA damage there were deregulated proteins which have no reported association with chemotherapy response or extensively reported link with solid malignancies. Those with no known association with chemotherapy or chemoresistance include heterogenous nuclear ribonucleoprotein A/B (HNRNPAB), nuclear distribution gene C (NUDC), TP5313, transferrin receptor 1 (TFRC), FDXR, and PAPSS2. The only protein found to have no known role in lung cancer or other solid malignancies was AARS, as mentioned previously. Of these proteins, only TP53I3 had prognostic potential whereas each of these proteins were identified as potentially targetable. Although further analysis is required to determine the precise role of these proteins, it remains possible that targeting these proteins alongside chemotherapy might enhance platinum agent activity.

Our exploratory study has applied a SWATH-MS proteomic based approach to identify proteins deregulated following cisplatin exposure which might serve as potential biomarkers or therapeutic targets in NSCLC. It is worth noting that our proteomics approach was not able to detect an entire proteome. Moreover, another limitation of our study was the use of a single cell line for our proteomic analysis. Given that the H460 cell line is not representative of all non-squamous NSCLC, further proteomics analysis of laboratory models of disease or clinically relevant samples would strengthen our findings. One such approach might be to also incorporate the comparison of *in vitro* models and *ex vivo* patient tissues pre- and post-therapy, to validate our findings and further identify novel druggable proteins. However, we note that validation of our SWATH-MS by western blot analysis and usage of a wider cell line panel confirmed our single cell line proteomics findings. These data point to the possibility that we have identified a common cisplatin response network, at least for some of the identified significantly deregulated proteins. Nonetheless, our study provides a rationale for further investigation of these deregulated and potentially exploitable proteins. Indeed, while the findings of this study will not immediately impact clinical decision making, the work provides a platform and impetus for further investigation and future drug development. Analysis of further cohorts is also necessary to determine the predictive potential of the biomarkers identified in our study. Moreover, further research is required to confirm the therapeutic potential of the identified proteins and to develop small molecules to inhibit them. Overall, future inhibition of, or testing biopsies for these proteins could be useful to enhance platinum-based chemotherapy response and improve health outcomes for people impacted by NSCLC.

## Data Availability Statement

The proteomics data presented in the study are deposited in the Paranorma Public repository, ProteomeXchange ID: PXD024209 (https://urldefense.com/v3/__https://panoramaweb.org/lGlNod.url__;!!NVzLfOphnbDXSw!RlKUaKl0OWpX2gD9cUB6_1rO--VMvbjHMnHX7KSfhgqrwfhpkVfx6TIais4-k4mrV5Y$).

## Author Contributions

MA, KO’B, and DR conceived and designed the study. MA supervised the study. S-LR, KD, SB, KS, CM, CO’L, ES, EB, A-MB, and MA performed the experiments and interpreted the data. MG, MM, NG, and EB performed the in silico analyses and bioinformatics. S-LR, KD, and MA analyzed the data. DR, KO’B, and MA wrote the manuscript. All authors contributed to the article and approved the submitted version.

## Conflict of Interest

The authors declare competing financial interests; KO’B and DR are founders of CARP Pharmaceuticals. EB, DR, and KO’B are founders of Carpe Vitae Pharmaceuticals. MA, EB, KO’B, and DR are inventors on patent applications filed by Queensland University of Technology.

The remaining authors declare that the research was conducted in the absence of any commercial or financial relationships that could be construed as a potential conflict of interest.

## Supplementary Material

Supplementary Figure 1 StringDB network analysis of proteins identified by quantitative mass spectrometry as significantly deregulated following cisplatin exposure. Line thickness is indicative of confidence in evidence linking proteins within a network.

Supplementary Figure 2 Identification of TP53 by IPA analysis as the predicted top upstream regulator of differentially regulated protein.
