# Supplementary figures and images for: Identification of Proteins Deregulated by Platinum-Based Chemotherapy as Novel Biomarkers and Therapeutic Targets in Non-Small Cell Lung Cancer
Source: Front Oncol. 2021 Mar 11;11:615967. doi: 10.3389/fonc.2021.615967 (PMC7991912; doi:10.3389/fonc.2021.615967)

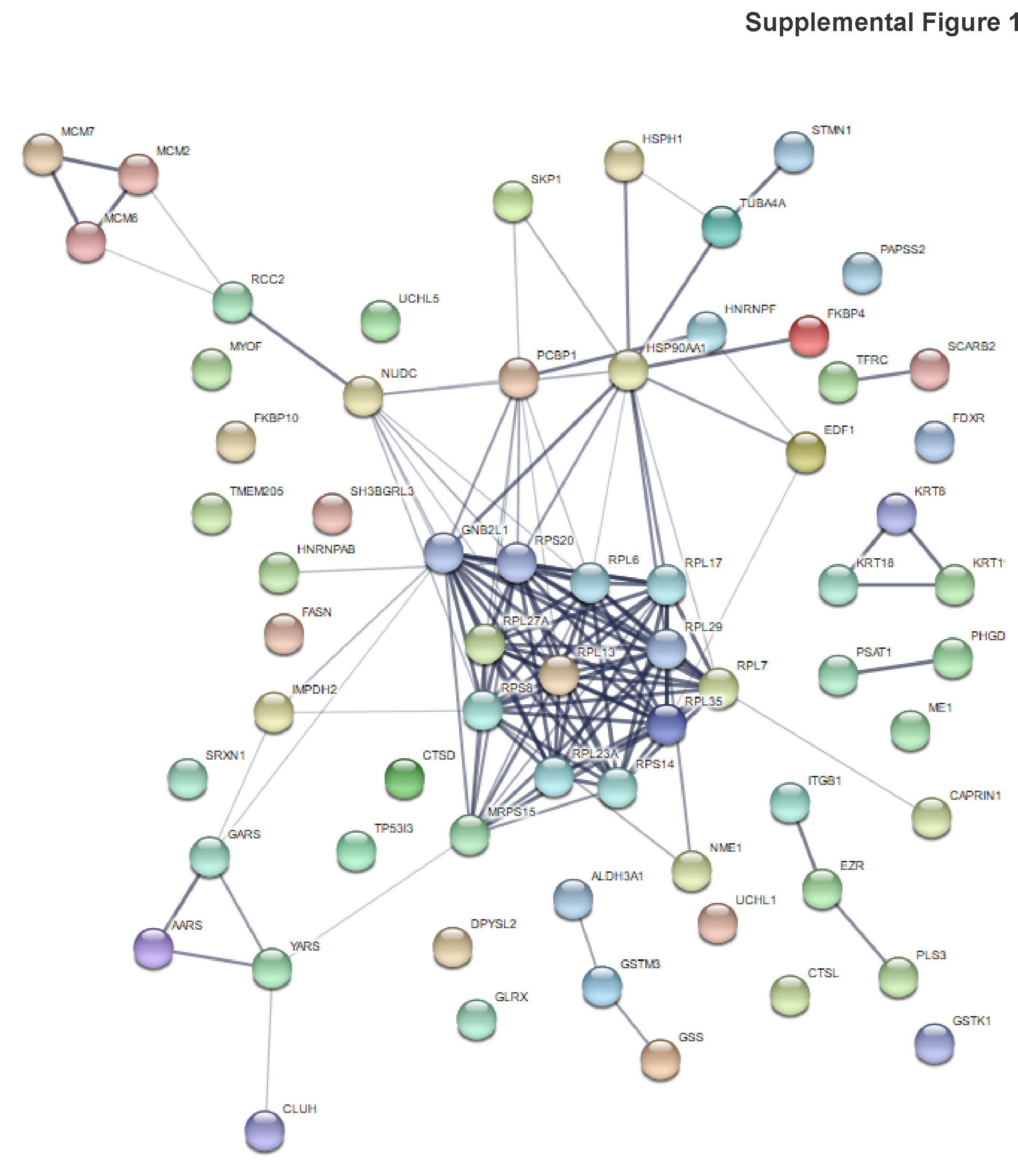

Supplement: Supplementary Figure 1 — StringDB network analysis of proteins identified by quantitative mass spectrometry as significantly deregulated following cisplatin exposure. Line thickness is indicative of confidence in evidence linking proteins within a network. [file Image_1.tif]

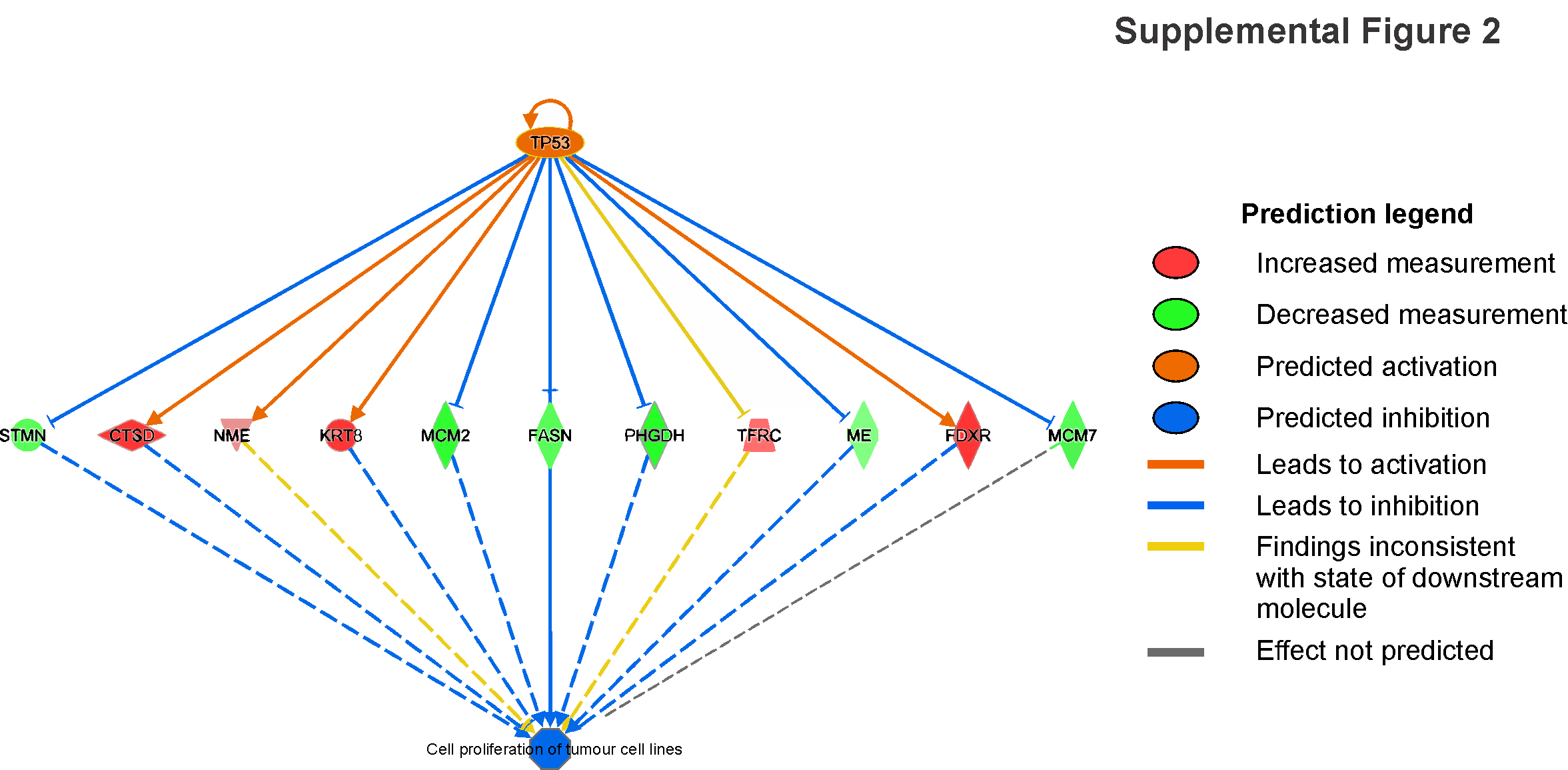

Supplement: Supplementary Figure 2 — Identification of TP53 by IPA analysis as the predicted top upstream regulator of differentially regulated protein. [file Image_2.tif]
